# Consistent 1,3-propanediol production from glycerol in mixed culture fermentation over a wide range of pH

**DOI:** 10.1186/s13068-016-0447-8

**Published:** 2016-02-06

**Authors:** Roman Moscoviz, Eric Trably, Nicolas Bernet

**Affiliations:** INRA, UR0050, Laboratoire de Biotechnologie de L’Environnement (LBE), Avenue des étangs, 11100 Narbonne, France

**Keywords:** 1,3-PDO, Metabolic patterns, Microbial consortia, Dark fermentation, Biodiesel

## Abstract

**Background:**

Glycerol is currently an over-produced chemical that can be used as substrate for the production of high value products such as 1,3-propanediol (1,3-PDO) in fermentation processes. The aim of this study was to investigate the effect of initial pH on a batch mixed culture fermentation of glycerol, considering both the bacterial community composition and the fermentation patterns.

**Results:**

For pH values between 5 and 9, 1,3-PDO production yields ranged from 0.52 ± 0.01 to 0.64 ± 0.00 ${\text{mol}}_{{ 1, 3{\text{-}} {\text{PDO}}}} {\text{mol}}_{\text{glycerol}}^{ - 1}$, with the highest values obtained at pH 7 and 8. An *Enterobacteriaceae* member closely related to *Citrobacter freundii* was strongly enriched at all pH values. Within the less dominant bacterial species, two different microbial community structures were found, one at acid pH values and another at neutral to basic pH values.

**Conclusions:**

1,3-PDO production was improved at pH values over 7. It was anti-correlated with lactate and ethanol production but positively correlated with acetate production. No direct correlation between 1,3-PDO production and a specific family of bacteria was found, suggesting functional redundancies in the microbial community. However, 1,3-PDO production yield remained high over the range of pH studied and was comparable to the best obtained in the same conditions in the literature.

**Electronic supplementary material:**

The online version of this article (doi:10.1186/s13068-016-0447-8) contains supplementary material, which is available to authorized users.

## Background

In order to reduce their fossil fuel dependency, several countries have favored the production of biofuels such as bioethanol or biodiesel. The European Union voted in 2009 a resolution to raise the share of EU energy consumption produced from renewable resources to 20 %, while reaching a 10 % share of renewable energy in the transport sector. Biodiesel is currently produced from transesterification of animal or vegetal oils. However, approximately 100 kg of glycerol are co-produced per ton of biodiesel produced [1]. This has led to an increase in world glycerol production over the last decade. This production reached about 3 million tons in 2011 and 4.7 million tons are expected to be produced in 2020 [2]. Therefore, it is a major issue to find a recycling solution for this glycerol to make the biodiesel production more sustainable.

Glycerol can be used as an inexpensive carbon substrate for fermentation to produce many economically interesting chemicals including 1,3-propanediol (1,3-PDO). 1,3-PDO is used for the production of solvents, cleaners, adhesives, resins, and cosmetics. It can also be used as a monomer for the production of polytrimethylene terephthalate (PTT) further used in textile industry [3]. Many micro-organisms from the *Enterobacteriaceae* and *Clostridiaceae* families are known as natural producers of 1,3-PDO from glycerol. So far, most studies about 1,3-PDO production from glycerol fermentation have focused on the use of pure cultures such as *Clostridium butyricum* [4] or *Klebsiella pneumoniae* [5]. High yields, productivities, and final 1,3-PDO concentrations have been achieved with pure cultures which require sterile conditions and the use of yeast or meat extract in the culture medium. To reduce the production costs, only few articles have reported the use of mixed cultures to convert crude glycerol from biodiesel production into 1,3-PDO under non-sterile conditions. Dietz et al. [6] successfully used mixed cultures from municipal wastewater treatment plant and reached yields between 0.56 and 0.76 mol_1,3-PDO_${\text{mol}}_{{{\text{glycerol}} }}^{{{ - 1} }}$ with a minimal culture medium containing crude glycerol. These production yields were slightly higher than the theoretical maximum yield of 0.72 mol_1,3-PDO_${\text{mol}}_{{{\text{glycerol}} }}^{{{ - 1} }}$ [6] because of the impurities contained in crude glycerol that could be used as additional substrates. Selembo et al. [7] and Liu et al. [8] achieved 1,3-PDO production yields close to the theoretical maximum (resp. 0.69 and 0.65 mol_1,3-PDO_${\text{mol}}_{{{\text{glycerol}} }}^{{{ - 1} }}$) when using mixed culture on glycerol fermentation.

Previous reported results using mixed cultures were obtained in different experimental conditions and, in particular, with pH values ranging from 5.5 to 8 and with different sources of glycerol [6–10], making difficult to outline the effects of pH. As reported by Samul et al. [11], the effects of crude glycerol impurities on the fermentation patterns can substantially vary, depending on their composition and the source of micro-organisms. The aim of this work was to investigate the effect of initial pH on batch production of 1,3-PDO under non-sterile conditions using a mixed culture as inoculum. Hence a minimal culture medium containing only pure glycerol with no additives such as yeast extract was used in order to reduce the sources of variability other than pH.

## Methods

### Inoculum

The microbial inoculum used in this work was a mixed culture issued from a long-term continuous dark fermentation lab-scale reactor operated at pH 6.5 under micro-aerobic conditions for the production of H_2_ from glycerol [12]. It was stored at 4 °C for 1 month before use.

### Fermentation medium

The composition of the fermentation medium (per liter of water) was modified from Dietz et al.'s as follows: 1.66 g glycerol, 1 g NH_4_Cl, and 0.5 g NaCl for pH-buffered experiments or 23.50 g glycerol, 2.5 g NH_4_Cl and 1.0 g NaCl for pH-regulated experiments (Sigma-Aldrich, ≥99 %). In all experiments, 20 mL of a trace element solution (1.5 g/L nitrilotriacetic acid; 3.0 g/L MgSO_4_·7H_2_O; 0.50 g/L MnSO_4_·H_2_O; 1.0 g/L NaCl; 0.10 g/L FeSO_4_·7H_2_O; 0.18 g/L CoSO_4_·7H_2_O; 0.10 g/L CaCl_2_·2H_2_O; 0.18 g/L ZnSO_4_·7H_2_O; 0.01 g/L CuSO_4_·5H_2_O; 0.02 g/L KAl(SO_4_)_2_·12H_2_O; 0.01 g/L H_3_BO_3_; 0.01 g/L Na_2_MoO_4_·2H_2_O; 0.03 g/L NiCl_2_·6H_2_O; 0.30 mg/L Na_2_SeO_3_·5H_2_O; 0.40 mg/L Na_2_WO_4_·2H_2_O) and 150 mM phosphate buffer were added.

### pH-buffered fermentation set-up

Batch experiments were performed in triplicates in glass bottles containing 200 mL of solution and around 300 mL of headspace. Bottles were sealed with butyl rubber septa and aluminum caps. Initial biomass was obtained after centrifugation of 33 mL of the inoculum (volatile solids = 0.40 ± 0.01 %_total mass_) at 12,000*g* for 15 min. The pellet was then suspended in the culture medium. Anoxic conditions were assured just after inoculation by flushing the media with high-purity N_2_ (>99.995 %) for at least 30 min. The temperature was controlled at 37 °C. Initial pH was adjusted at 4, 5, 6, 7, 8, 9, or 10 using 150 mM phosphate buffer and hydrochloric acid. Final pH values were, respectively, 3.9 ± 0.2, 4.2 ± 0.2, 5.7 ± 0.2, 6.9 ± 0.1, 7.7 ± 0.2, 8.0 ± 0.2, and 9.9 ± 0.2.

### pH-regulated fermentation set-up

Glycerol fermentations under pH regulation were conducted in four replicates in glass reactors containing 1 L of solution and about 500 mL of headspace. The temperature was controlled at 37 °C and the pH was regulated at 7.0 by adding 2 M NaOH (pH probe InPro 4260i, Mettler Toledo). Bottles containing pH 7 from the pH-buffered experiments were used as inoculum after storage at 4 °C. Initial biomass was obtained after centrifugation of 100 mL of the inoculum at 12,000*g* for 15 min. The pellet was then suspended in the culture medium. Anaerobic conditions were assured just after inoculation by flushing the media with high-purity N_2_ (>99.995 %) for at least 30 min.

### Analytical methods

Concentrations of glucose, glycerol, 1,3-PDO, and organic acids were measured by HPLC with a refractive index detector (Waters R410). Samples were first centrifuged at 12,000*g* for 15 min and then supernatants were filtered with 0.2 µm syringe filters. HPLC analysis was performed at a flow rate of 0.4 mL/min on an Aminex HPX-87H, 300 × 7.8 mm (Bio-Rad) column at a temperature of 35 °C. H_2_SO_4_, 4 mM was used as the mobile phase. Biogas composition was determined using a gas chromatograph (Clarus 580, Perkin Elmer) equipped with a thermal conductivity detector. The columns used were a RtQbond column (for H_2_, O_2_, N_2_, and CH_4_) and a RtMolsieve column (for CO_2_), and the gas vector was argon at a pressure of 3.5 bar.

The COD balances were established based on the number of electrons per mol of each fermentation product and for microbial biomass, assuming an elemental composition of C_4_H_7_O_2_N [13]. Biomass was estimated from the metabolites produced considering a* Y*_X/ATP_ of 10.5 g/mol [14].

### Microbial community analysis

DNA was extracted with the QIAamp fast DNA stool mini kit in accordance with the manufacturer’s instructions (Qiagen, Hilden, Germany). Extractions were confirmed using Infinite 200 PRO NanoQuant (Tecan Group Ltd., Männedorf, Switzerland). The V4 and V5 regions of the 16S rRNA genes were amplified using the primers 515F (5′-GTGYCAGCMGCCGCGGTA-3′) and 928R (5′-CCCCGYCAATTCMTTTRAGT-3′), which captures most of the bacterial and archaeal diversity [15]. Adapters were added for multiplexing samples during the second amplification step of the sequencing. The PCR mixtures (50 µl) contained 0.5 U of Pfu Turbo DNA polymerase (Stratagene) with its corresponding buffer, 200 mM of each dNTP, 0.5 mM of each primer, and 10 ng of genomic DNA. Reactions were performed in a Mastercycler thermal cycler (Eppendorf) as follows: 94 °C for 2 min, followed by 35 cycles of 94 °C for 1 min, 65 °C for 1 min, and 72 °C for 1 min, with a final extension at 72 °C for 10 min. The amount and size of PCR products were determined using a Bioanalyzer 2100 (Agilent). A capillary electrophoresis single-strand conformation polymorphism (CE-SSCP) method was used for PCR product's diversity characterization. Samples were heat-denatured at 95 °C for 5 min and re-cooled directly in ice for 5 min. CE-SSCP electrophoresis was performed in an ABI Prism 3130 genetic analyzer (Applied Biosystems) in 50 cm capillary tubes filled with 10 % glycerol, conformation analysis polymer and corresponding buffer (Applied Biosystems). Samples were eluted at 12 kV and 32 °C for 30 min, as described elsewhere [16]. CE-SSCP profiles were aligned with an internal standard (ROX) to consider the inter-sample electrophoretic variability. CE-SSCP profiles were normalized using the StatFingerprints library [17] in R software version 2.9.2 (R. Development Core Team 2010). The community composition was also evaluated using the MiSeq v3 chemistry (Illumina) with 2 × 300 bp paired-end reads at the GenoToul platform (www.genotoul.fr). Sequences were retrieved after demultiplexing, cleaning, and affiliating sequences using mothur [18]. Sequences have been submitted to GenBank with accession No. KT287117–KT288056.

### Quantitative PCR (qPCR)

PCRs were prepared using 96-well real-time PCR plates (Eppendorf, Hamburg, Germany) and Mastercycler ep gradient S (Eppendorf, Hamburg, Germany). Then, 6.5 μl of Express qPCR supermix with premixed ROX (Invitrogen, France), 2 μl of DNA extract with three appropriate dilutions, 100 nM forward primer F338-354 (5′-ACTCC TACGG GAGGC AG-3′), 250 nM reverse primers R805-785 (5′-GACTA CCAGG GTATC TAATC C-3′), 50 nM TaqMan probe, and water were added to obtain a final volume of 12.5 μl for all analyses.

An initial incubation of 2 min at 95 °C and 40 cycles of denaturation (95 °C, 7 s; 60 °C, 25 s) were performed. One standard curve was generated from each assay by using tenfold dilutions in sterilized water (Aguettant Laboratory, Lyon, France) of a target plasmid (Eurofins Genomics, Germany). The initial DNA concentrations were quantified using the Infinite 200 PRO NanoQuant (Tecan, France). The average number of bacterial cells was estimated by dividing the average number of 16S rRNA gene copies per cell by a factor 4.1 [19].

### Theoretical yield calculations

Metabolic pathways of glycerol fermentation were assumed to be similar as in [20]. In particular, the biochemical routes leading to lactate, acetate, and ethanol without formate production were written as follows:

$${\text{Glycerol}} + {\text{ADP}} + {\text{P}}_{\it{i}} + {\text{NAD}}^{+} \to {\text{Lactate}} + {\text{ATP}} + {\text{H}}_{2} {\text{O}} + {\text{NADH}}_{2}$$

$${\text{Glycerol}} + 2\left( {{\text{ADP}} + {\text{P}}_{{i}} } \right) + 3 {\text{NAD}}^{+} \to {\text{ Acetate}} + \text{{CO}}_{ 2} + 2{\text{ATP}} + {\text{H}}_{ 2} {\text{O}} + 3 {\text{NADH}}_{ 2}$$

$${\text{Glycerol}} + {\text{ADP}} + {\text{P}}_{{i}} +{\text{NAD}}^{+} \to {\text{Ethanol}} + {\text{CO}}_{2} +{\text{ATP}} + {\text{H}}_{ 2} {\text{O}} + {\text{NADH}}_{ 2}$$

$${\text{Glycerol }} + {\text{ NADH}}_{ 2} \to \, 1,3{\text{-}}{\text{propanediol }}+ {\text{NAD}}^{ + } +{\text{ H}}_{ 2} {\text{O}}.$$

The conversion of formate into hydrogen was assumed as follows:

$${\text{Formate}} + {\text{H}}_{ 2} {\text{O}}_{{}} \to {\text{ HCO}}_{ 3}^{-} +{\text{ H}}_{ 2}$$

The elemental constitution of biomass was assumed to be C_4_H_7_O_2_N with a biomass production yield of 10.5 g/mol_ATP_ [14], leading to the following equation:

$$4_{{}} {\text{Glycerol}} + 3 {\text{NH}}_{3} + 30{\text{ATP}}+24 {\text{H}}_{ 2} {\text{O}} + 4 {\text{NAD}}^{ + }_{{}} \to_{{}} 3 {\text{ C}}_{ 4} {\text{H}}_{ 7} {\text{O}}_{ 2} {\text{N}} + 4 {\text{NADH}}_{ 2} +30 \left( {{\text{ADP}} + {\text{P}}_{{i}} } \right)$$

### Pearson correlation matrix

A Pearson correlation matrix was calculated from metabolite profiles after 3 days of fermentation (*n* = 15) and the bacterial community composition obtained after sequencing (*n* = 5, only one per triplicate). The correlation and significance calculations were made with the R 3.1.3 software (R Development Core Team 2010) and the function “rcorr” of the package Hmisc. The hierarchical clustering was made with the function “corrplot” of the package corrplot using the centroid method.

### Principal component analysis (PCA)

In order to analyze and compare the microbial consortia, a principal component analysis (PCA) was performed on the microbial community compositions obtained from CE–SSCP with the R 2.12 software (R Development Core Team 2010), the vegan 2.12.2 package.

## Results

### Effect of pH on fermentation products

**Fig. 1:**
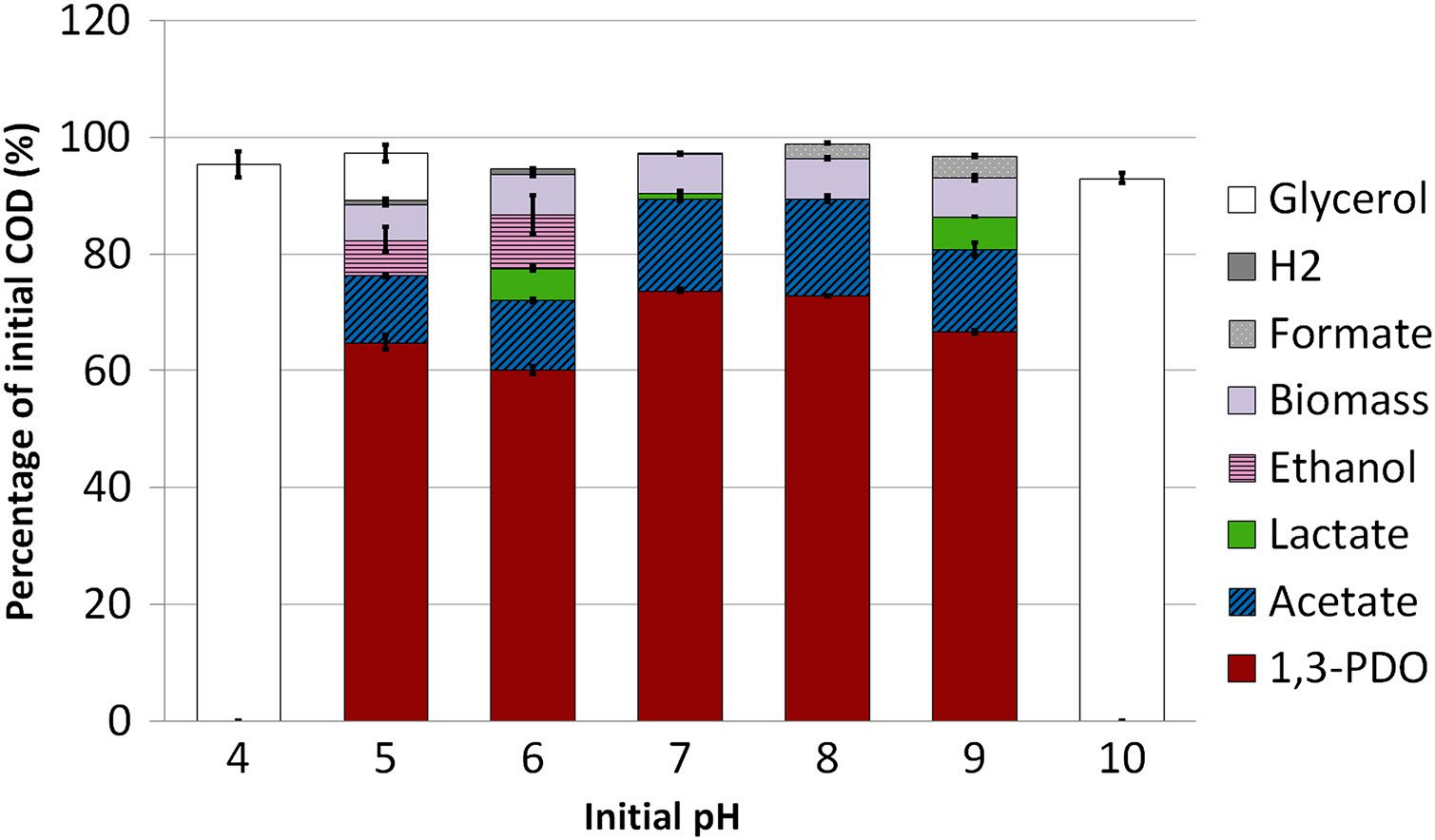
COD balances calculated from the metabolites measured after 3 days of fermentation in triplicate experiments in pH-buffered reactors. Results are normalized on initial COD. The biomass was estimated from the ATP production associated to the different metabolites production

To evaluate the effect of initial pH on glycerol fermentation by a mixed culture, a range of initial pH values between 4 and 10 was investigated in batch reactors. A low initial concentration of 1.66 g of glycerol was used to avoid a pH drop during fermentation. COD mass balances are shown in Fig. 1 (more details on COD mass balances are presented in Additional file 1). COD mass balance closed between 93 and 102 %, indicating that no major metabolic by-product was missed during the batch fermentation. After 3 days of fermentation, glycerol was depleted in most of the reactors except those running at the extreme pH 4, 5, and 10 with 95.4, 8.1, and 93.0 % of the initial glycerol remaining, respectively. It was assumed that no fermentation occurred at pH 4 and 10. For all other pH values, the main metabolite produced was 1,3-PDO (60–74 %_total COD_) with acetate as major by-product (11–17 %_total COD_). The 1,3-PDO production yields ranged from 0.52 ± 0.01 to 0.64 ± 0.00 mol_1,3-PDO_${\text{mol}}_{{{\text{glycerol}} }}^{{{ - 1} }}$. The best values were obtained at pH 7 and 8 and corresponded to 90 % of the maximum theoretical yield of 0.72 mol_1,3-PDO_${\text{mol}}_{{{\text{glycerol}} }}^{{{ - 1} }}$ [6] with a final concentration of 0.86 ± 0.00 g/L. Ethanol was only produced for pH values below 6 (6–9 %_total COD_), while acetate production decreased. At pH values over 7, formate production increased from 0 to 9 %_total COD_. H_2_ was only detected for pH values below 7 and represented less than 1 % of the total COD. Methane was not detected in any condition, which was not surprising since the initial inoculum originated from an output of a continuous reactor in which methanogenesis did not occur (low HRT). Although basic pH around 7–8 may favor the emergence of methanogens in long-term operation of the reactor, several studies reported that high 1,3-PDO final titers were obtained at pH between 5 and 6 [21], and pH 8 [7] without methane production.

### Comparison with theoretical yields

**Fig. 2:**
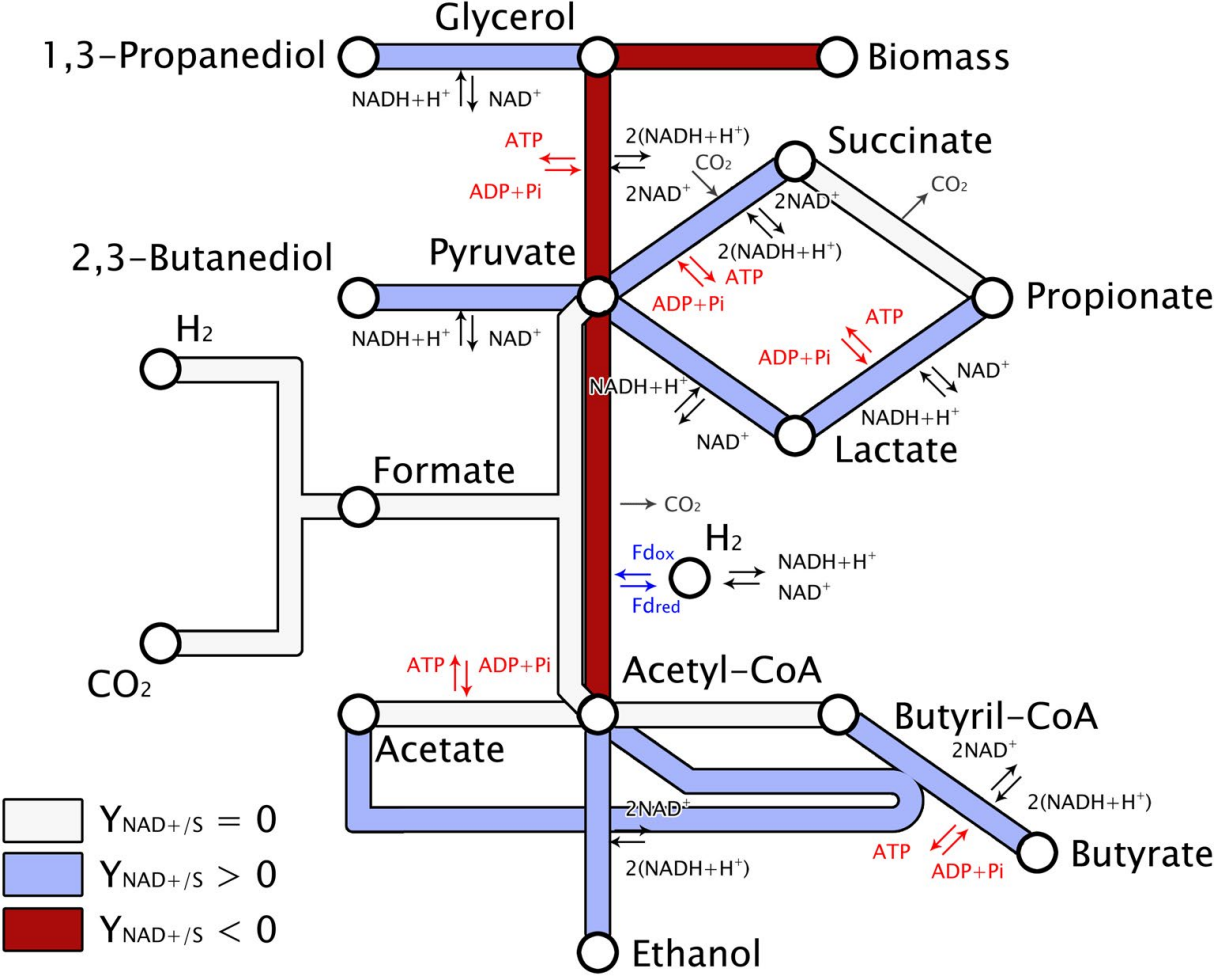
Simplified catabolic pathways of glycerol fermentation. Fd_ox_ and Fd_red_ stand for the oxidized and reduced form of ferredoxin, respectively. Adapted from [20]

**Fig. 3:**
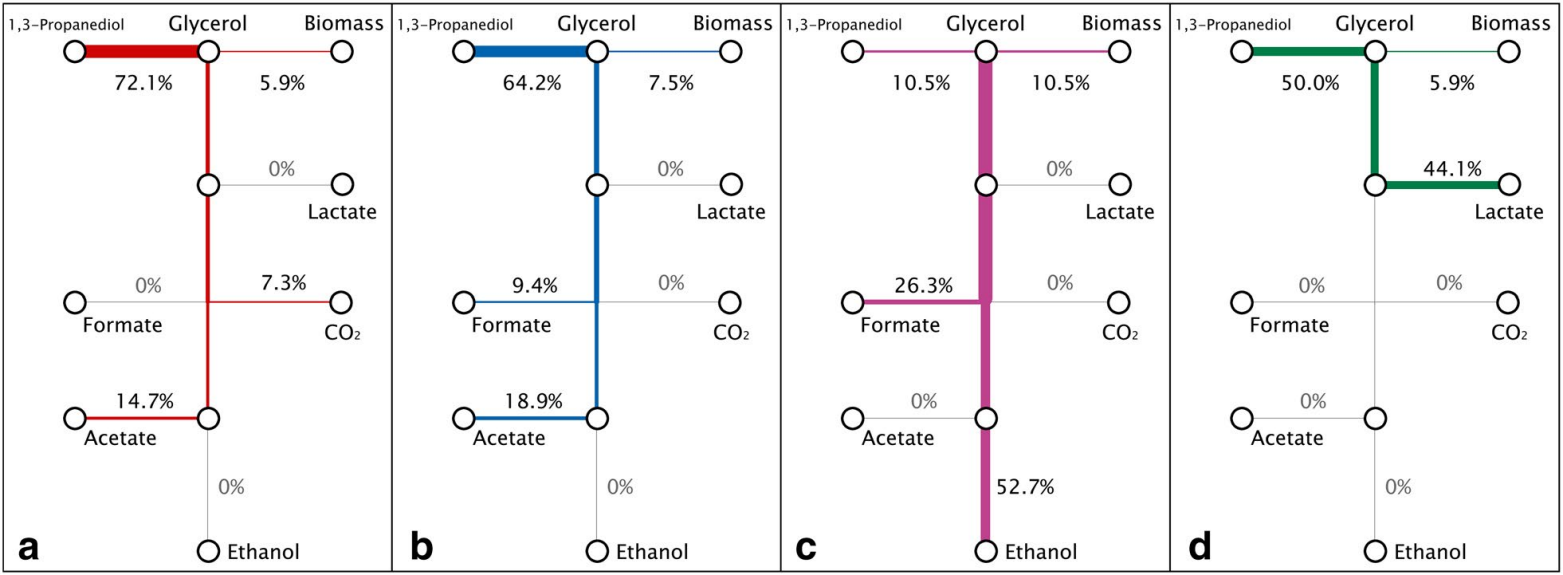
Carbon flux trees according to theoretical pathways. **a** Maximal 1,3-propanediol production. **b** Acetate and Formate pathways. **c** Maximal growth yield. **d** Minimal growth yield. The values in percentage represent the proportion of initial carbon that is found in the final products

Metabolic pathways of glycerol fermentation are well known and have been described in many studies. A simplified representation is provided in Fig. 2. In order to find the global reactions leading to (i) maximal 1,3-PDO production (ii) maximal biomass growth, and (iii) minimal biomass growth, the following redox and ATP balanced reactions were calculated by aggregating the equations of glycerol metabolism as provided in the material and method section and presented in Fig. 3:

1 $$68 {\text{ Glycerol}} + 3 {\text{ NH}}_{ 3} \to 3{\text{ C}}_{ 4} {\text{H}}_{ 7} {\text{O}}_{ 2} {\text{N}} + 15 {\text{ Acetate}} + 15 {\text{ CO}}_{ 2} + 49\,\, 1,3{\text{-}} {\text{PDO}} + 40 {\text{ H}}_{ 2} {\text{O}}$$

2 $$53 {\text{ Glycerol}} + 3 {\text{ NH}}_{ 3} \to 3{\text{ C}}_{ 4} {\text{H}}_{ 7} {\text{O}}_{ 2} {\text{N}} + 15 {\text{ Acetate}} + 15 {\text{ Formate}} + 34\,\, 1,3{\text{-}} {\text{PDO}} + 25{\text{ H}}_{ 2} {\text{O}}$$

3 $$38 {\text{ Glycerol}} + 3{\text{ NH}}_{ 3} \to 3{\text{ C}}_{ 4} {\text{H}}_{ 7} {\text{O}}_{ 2} {\text{N}} + 30{\text{ Ethanol}} + 30{\text{ Formate}} + 4 \,\,1,3 {\text{-}} {\text{PDO}} + 10 {\text{ H}}_{ 2} {\text{O}}$$

4 $$6 8 {\text{ Glycerol}} + 3{\text{ NH}}_{ 3} \to 3 {\text{ C}}_{ 4} {\text{H}}_{ 7} {\text{O}}_{ 2} {\text{N}} + 30{\text{ Lactate} }+ 34 \,\,1,3 {\text{-}} {\text{PDO}} + 40{\text{ H}}_{ 2} {\text{O}}$$

The maximal theoretical production yield of 1,3-PDO (0.72 mol/mol) could be obtained when only acetate was produced, according to Eq. (1). The theoretical maximal growth was reached when ethanol was produced together with formate as in the Eq. (3), leading to a minimal 1,3-PDO yield of 0.11 mol/mol. The theoretical biomass growth was minimal if only lactate and acetate were produced (Eqs. (1) and (4)) but the production of lactate had a negative impact on 1,3-PDO production. The production of formate together with acetate had also a negative impact on 1,3-PDO (Eq. (2)). These theoretical values have been compared to the actual values obtained at different pH values and are shown in Table 1. The best 1,3-PDO production values were obtained at pH 7 and 8 and were close to those obtained with Eq. (4) (i.e., *Y*_Acetate/S_ = 0.28 mol/mol and *Y*_PDO/S_ = 0.64 mol/mol) but with much less formate or hydrogen produced, maybe due to measurement errors in hydrogen production.

**Table 1:** Comparison of the experimental yields obtained in this study with theoretical yields calculated considering anabolism and catabolism

	Theoretical values				Experimental values				
	Maximal PDO production	Minimal growth yield	Maximal growth yield	Acetate and formate pathway	pH 5	pH 6	pH 7	pH 8	pH 9
*Y*_X/S_ (g/mol)^a^	4.45	4.45	7.97	5.72	5.39 ± 0.25	6.18 ± 0.62	5.90 ± 0.14	6.15 ± 0.41	5.87 ± 0.80
*Y*_ATP/S_ (mol/mol)^b^	0.44	0.44	0.79	0.57	0.51 ± 0.02	0.59 ± 0.06	0.56 ± 0.01	0.59 ± 0.04	0.56 ± 0.07
*Y*_PDO/S_ (mol/mol)	0.72	0.50	0.11	0.64	0.61 ± 0.04	0.52 ± 0.01	0.64 ± 0.00	0.63 ± 0.01	0.58 ± 0.01
*Y*_Acetate/S_ (mol/mol)	0.22	0	0	0.28	0.21 ± 0.02	0.21 ± 0.01	0.27 ± 0.01	0.29 ± 0.02	0.25 ± 0.04
*Y*_Ethanol/S_ (mol/mol)	0	0	0.79	0	0.07 ± 0.05	0.11 ± 0.07	0	0	0
*Y*_Lactate/S_ (mol/mol)	0	0.44	0	0	0	0.06 ± 0.01	0.01 ± 0.01	0	0.07 ± 0.00
*Y*(_Formate+H2)/S_ (mol/mol)	0	0	0.79	0.28	0.06 ± 0.01	0.07 ± 0.00	0.01 ± 0.01	0.17 ± 0.01	0.26 ± 0.02

^a^The biomass yield was calculated assuming an elemental composition of C_4_H_7_O_2_N [14] and that all the ATP produced was used for biomass production

^b^The ATP yield was calculated from the metabolites measured after 3 day of fermentation:* Y*_ATP/Acetate_ = 2; *Y*_ATP/Ethanol_ = 1; *Y*_ATP/Lactate_ = 1; * Y*_ATP/PDO_ = 0

### Microbial communities and growth

Biomass was estimated after 3 days of fermentation from qPCR on total bacterial DNA. The low initial biomass concentration of 5.9 ± 1.7 × 10^5^ bact/mL after inoculation could explain the long lag phase observed at all pH values. The final biomass concentration ranged between 10^8^ and 10^9^ bact/mL in all reactors in which glycerol fermentation occurred, except for the reactors running at pH 9 (7.4 ± 1.3 × 10^6^ bact/mL). This value obtained at pH 9 is very low compared to the biomass estimated with ATP production. This could be due to ATP dissipation for maintaining intracellular pH at 7. Therefore, it was clear that bacterial growth was strongly inhibited at extreme pH values lower than 5 and above 8.

**Fig. 4:**
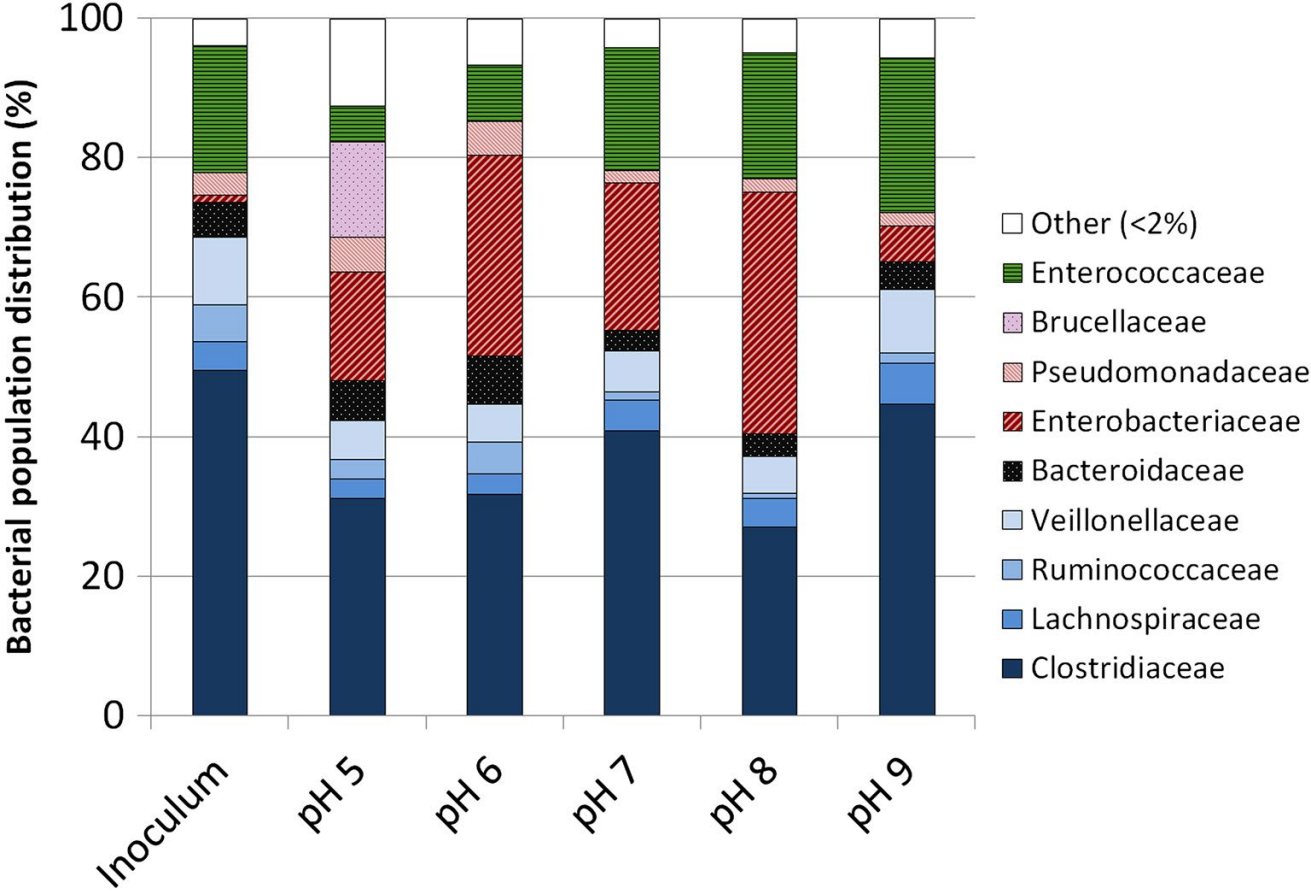
Bacterial population distribution within the taxonomic families of the inoculum and after 3 days of fermentation in pH-buffered reactors at different pH values. This distribution is based on 16S rRNA genes identification retrieved from MiSeq sequencing. Other stand for the families containing less than 2 % of the total bacterial populations

To observe the effect of pH on microbial communities, MiSeq sequencing was performed on the inoculum and on samples after 3 days of fermentation (Fig. 4). The inoculum was mainly composed of bacteria from the *Clostridiaceae* and *Enterococcaceae* families (resp. 50 and 18 % of 82,243 sequences). Two OTUs were dominant, one in each family, and represented 46 % and 18 % of the total bacterial community. Nucleotide sequence analyses of their 16S rRNA genes revealed resp. 99 and 100 % of sequence homology with *Clostridium intestinale* and *Enterococcus cecorum*. *C. intestinale* is known to be an aerotolerant species, able to grow on glycerol and to produce H_2_ [22–24], which is consistent with the inoculum origin. After 3 days of fermentation, the bacterial community observed at pH 9 was very close to the inoculum, probably because there was practically no bacterial growth. For every other pH condition, an *Enterobacteriaceae* species was enriched whose 16S rRNA gene had 100 % of sequence homology with *Citrobacter freundii*, a species studied for 1,3-PDO production from glycerol [25, 26]. A *Brucellaceae* species which had 100 % similarity with *Ochrobactrum anthropi* was also favored at pH 5.

### Correlations between microbial community and fermentation patterns

**Fig. 5:**
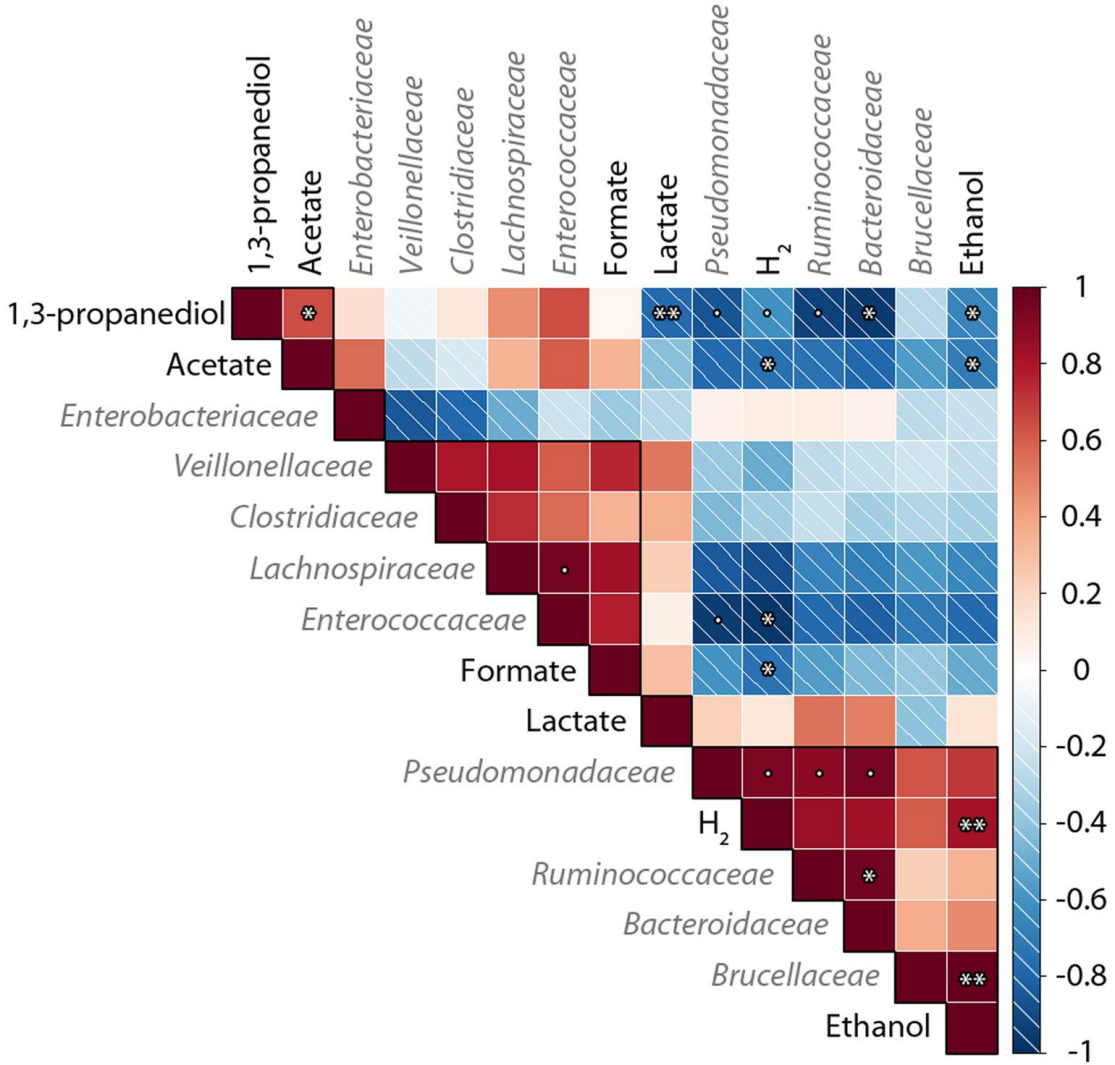
Pearson correlation matrix calculated from metabolite production profiles and sequencing results after 3 days of fermentation. The hatched squares correspond to negative correlations and the full squares to positive correlations. The *black outlines* are the result of hierarchical clustering for n = 5 groups. p-values: ** ≤0.001; *≤0.01; •≤0.05

In order to highlight correlations between the composition of microbial communities and fermentation patterns, a Pearson correlation matrix was calculated with the bacterial families and metabolites produced as variables (Fig. 5). 1,3-PDO was found to be positively correlated to acetate (*r* = 0.64, *p* ≤ 0.01) and negatively correlated to lactate (*r* = –0.78, *p* ≤ 0.001), ethanol (*r* = –0.65, *p* ≤ 0.01), and hydrogen (*r* = –0.60, *p* ≤ 0.05). It was also negatively correlated to the emergence of bacteria from the *Pseudomonadaceae* (*r* = –0.85, *p* ≤ 0.05), *Ruminococcaceae* (*r* = –0.92, *p* ≤ 0.05), and *Bacteroidaceae* (*r* = –0.96, *p* ≤ 0.01) families. A hierarchical cluster analysis on the Pearson correlation matrix also highlighted two groups of bacteria. The first one was composed of bacteria from *Veillonellaceae*, *Clostridiaceae*, *Lachnospiraceae*, and *Enterococcaceae* families and was linked with formate production. The second one was composed of bacteria from *Pseudomonadaceae*, *Ruminococcaceae*, *Bacteroidaceae,* and *Brucellaceae* and linked with ethanol and hydrogen production. There was a high positive correlation between ethanol and the presence of *Brucellaceae* bacteria (*r* = 0.99, *p* ≤ 0.001), and hydrogen production and the presence of *Pseudomonadaceae* bacteria (*r* = 0.93, *p* ≤ 0.05). Lactate was not found to be correlated to a specific group of bacteria.

### pH-regulated fermentations

To see whether the performances obtained with a low substrate concentration were still valid at higher substrate load, assays were carried out in batch mode in pH-regulated reactors at an initial glycerol concentration of 23.5 g/L. A pH of 7.0 was selected to regulate the fermenters since it was the condition that led to the best 1,3-PDO yield during the pH-buffered assays. The fermentation started after a 19 h lag phase, probably due to the inoculum storage and all the substrates were then depleted within 11.5 h. The COD mass balance was close at 95 % with 1,3-PDO as the major product (61 %_total COD_) (more details on metabolites distribution are presented in Additional file 2). The 1,3-PDO yield and productivity were, respectively, 0.53 ± 0.02 mol_1,3-PDO_${\text{mol}}_{{{\text{glycerol}} }}^{{{ - 1} }}$ and 0.89 ± 0.02 g/L h, and a final concentration of 10.3 ± 0.3 g/L was achieved. Major by-products were ethanol (11 %_total COD_), acetate (7 %_total COD_), and lactate (7 %_total COD_). Ethanol was mainly produced within the first 4 hours of fermentation. Formate and succinate were also produced in small quantities (resp. 2 %_total COD_ and 1 %_total COD_).

## Discussion

### Effect of pH on microbial populations

**Fig. 6:**
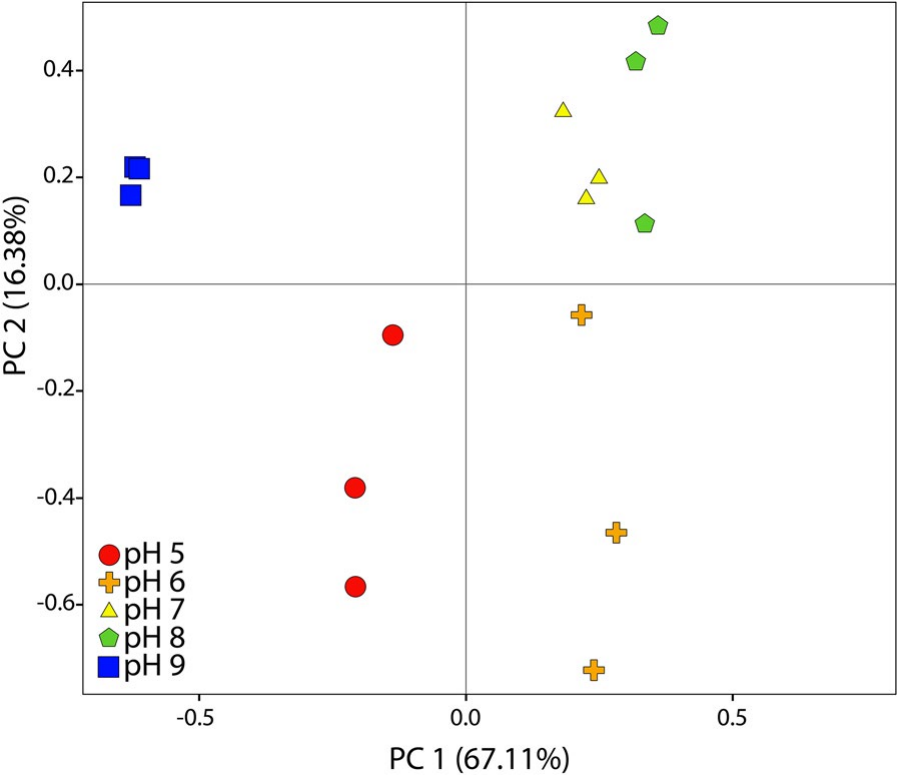
PCA performed on the composition of bacterial communities obtained with CE-SSCP after 3 days of fermentation in pH-buffered reactors

In order to compare the bacterial populations obtained at the end of the fermentation with the different pH values, a PCA was performed (Fig. 6). Most of the total variance (67.1 %) was explained by the principal compound 1 (PC 1) that was able to discriminate samples between neutral pH from 6 to 8 and extreme pH values of 5 and 9. This PC was supported by the emergence of the *Enterobacteriaceae* species and the decrease of the *Clostridiaceae* species that were predominant in the inoculum. Surprisingly, these two predominant families were found to have non-significant and low correlations with the metabolites produced suggesting that the differences found in the fermentation patterns were more related to less dominant species. It was shown that sub-dominant species in mixed culture fermentations can have significant effect on fermentation patterns and therefore have to be considered even at low abundance [27]. The PC 2 (16.4 % of total variance) separated the bacterial population observed at low pH (≤6) and neutral to basic pH (≥7). This PC separated the two groups highlighted by the hierarchical clustering of the correlation matrix. The growth of *Pseudomonadaceae*, *Ruminococcaceae*, *Bacteroidaceae,* and *Brucellaceae* species together with ethanol and H_2_ production was then found to occur at low pH (<6). On the other hand, the growth of the species from the *Enterococcaceae*, *Clostridiaceae*, *Lachnospiraceae*, and *Veillonellaceae* families, associated to formate production, was favored at high pH (≥7). The high pH microbial community was more favorable for 1,3-PDO than the one found for pH values below 6 in which many micro-organisms were strongly anti-correlated with 1,3-PDO production. However, no significant and direct link between a specific bacterial family and a better 1,3-PDO has been found. It was also found that lactate was neither correlated to a specific bacterial family nor to pH conditions.

### pH-induced H_2_/formate shift

It is usual to observe H_2_ production from glycerol or glucose fermentation depending strongly on the initial pH. The shift from formate to H_2_ production observed in this study when pH decreased was previously described by Temudo et al. [28] who used a mixed culture for glucose fermentation. It was observed during this study that the hydrogen/formate molar ratio decreased concomitantly with the increase initial pH values. Considering the following equation and its Gibbs free energy [28]:

$${\text{Formate}} + {\text{H}}_{2} {\text{O}} \to {\text{HCO}}_{3}^{ - } + {\text{H}}_{2} \quad \Delta {\text{G}}^\circ {^\prime} = 1.3\,{\text{kJ/mol}}$$

The observed shift from formate to H_2_ could be explained by thermodynamic considerations. This reaction is very close to the thermodynamic equilibrium and is catalyzed by the formate hydrogen lyase complex that is reversible. As the p*K*_a_ value of carbonate is 6.37 (at 25 °C), a pH increase above this value would favor carbonate accumulation in the bulk and therefore inhibit formate splitting into carbonate and H_2_. Considering that neither methanogenesis nor acetogenesis is occurring, a low H_2_ production could mean that formate is produced and/or NADH_2_ is formed from ferredoxin (see Fig. 2). However, it is very likely that hydrogen was underestimated during this study when comparing the metabolic profiles obtained for pH values between 5 and 7 and theoretical values (see Table 1).

### Ethanol production

From a theoretical analysis of all the possible glycerol fermentation pathways, it is clear that the acetate pathway leads to the highest 1,3-PDO production. In this study, a shift in acetyl-CoA derived product was observed from acetate to ethanol at pH values below 6 with an expected decrease of the 1,3-PDO production yields. From a thermodynamic point of view, Rodriguez et al. [29] showed in their metabolic-based model that for pH values below 5.6, ethanol is the metabolite that is generating the maximum energy for growth. Their calculation considers the energetic cost of acid transportation through the cellular membrane. At pH lower than 5.6, the energetic cost becomes more important than the energy supplied to the metabolism by the extra ATP produced during acetate production. Therefore, ethanol is energetically favored over acetate at low pH values. However, the ethanol shift cannot be only explained by energetic reasons and seems to be also strain-dependent. *Klebsiella variicola* has been reported to produce ethanol from glycerol with high yields at pH values ranging from 8 to 9 [30]. Temudo et al. [9] also showed ethanol production from glycerol at pH 8 from a mixed culture dominated by an *Enterobacteria* species close to *Klebsiella oxytoca*. In addition, *Clostridium acetobutylicum*, a bacterium used for acetone–butanol–ethanol production, is known for switching its metabolism from acidogenesis to solventogenesis when external pH drops under 5 [31]. In this study, ethanol production was highly correlated with *Brucellaceae* species and was only found when pH was below 6.

### Towards high 1,3-PDO concentrations

Initial high 1,3-PDO production yields were obtained at low glycerol concentration with a low impact of the pH. To determine whether such performances could be reached at higher substrate concentration, an assay was performed in pH-regulated batch reactors with an initial glycerol concentration of 23.5 g/L at pH 7. In this experiment, a 1,3-PDO yield of 0.53 ± 0.02 mol_1,3-PDO_${\text{mol}}_{{{\text{glycerol}} }}^{{{ - 1} }}$ was obtained, which is slightly lower but still consistent with the one obtained with the reactors buffered at pH 7 and with an initial substrate concentration of 1.66 g/L (0.64 ± 0.00 mol_1,3-PDO_${\text{mol}}_{{{\text{glycerol}} }}^{{{ - 1} }}$). Nevertheless, this yield is still high considering that a minimal medium with no vitamins or yeast extract was used. It is consistent with the results obtained by Dietz et al. in similar conditions with crude glycerol (yield of ~0.60 mol_1,3-PDO_${\text{mol}}_{{_{\text{glycerol}} }}^{ - 1}$ and productivity of ~1 g/L h) and by Kanjilal et al. with pure glycerol (0.52 mol_1,3-PDO_${\text{mol}}_{{{\text{glycerol}} }}^{{{ - 1} }}$) [6, 10]. These different results tend to show that mixed culture can be a viable option for 1,3-PDO production from pure or crude glycerol, even though two major challenges remain to sustain an efficient production of high concentration of 1,3-PDO. The first one is the use of crude glycerol issued from biodiesel production, which contains various impurities such as methanol and KOH at high concentrations [8, 10, 11, 32]. These impurities may have positive effects through the addition of carbon sources and nutriments that can be used by the micro-organisms and thus increase the 1,3-PDO production [6, 10, 11]. But methanol that is always present in these impurities can also inhibit the microbial growth, even at low concentration, and therefore decrease 1,3-PDO productivity and glycerol consumption [8, 32]. As crude glycerol composition may vary from a source to another, it is rather difficult to extend our conclusions when considering the combined effect of the impurities on glycerol fermentation. For that reason, mixed culture fermentation has the advantage to be more robust to environmental changes. The second challenge is to increase the final 1,3-PDO concentration, while keeping high productivities and production yields. A substrate inhibition has been reported at initial concentration higher than 70 g/L of crude glycerol for *C. butyricum* [33, 34]. This inhibition was also observed by Dietz et al. when mixed cultures were used [6]. Therefore, fed-batch process seems to be the best way to increase final 1,3-PDO concentration, while avoiding substrate inhibition. Using a fed-batch reactor with a continuous feed, mixed cultures and minimal medium, Dietz et al. obtained a final concentration of 70 g/L of 1,3-PDO with a yield of 0.56 mol_1,3-PDO_${\text{mol}}_{{{\text{glycerol}} }}^{{{ - 1} }}$ and a productivity of 2.60 g/L h [6]. Another interesting process named electro-fermentation showed promising results by reaching a final 1,3-PDO concentration of 42 g/L [35]. These results are outstanding considering that non-sterile conditions and minimal medium were used and are compared with the best performances obtained with pure culture [25].

## Conclusions

When considering the Pearson correlation matrix (Fig. 5) and the PCA results (Fig. 6), it appeared in this study that pH had a significant impact on both bacterial growth, the composition of the bacterial community and metabolic profiles. The predominant bacteria from *Clostridiaceae* and *Enterobacteriaceae* families could not explain alone the changes in metabolic profiles. Within the less dominant species, two different communities were found, one at acid pH values and another at neutral to basic pH values. The latter one was favorable to 1,3-PDO yield even if no significant correlation between a specific bacterial family of this community and a good 1,3-PDO yield was found. It was likely that there were a functional redundancy within this community. From the theoretical analysis of the metabolic pathways of glycerol fermentation (Table 1) and the correlation matrix (Fig. 5), it was clear that 1,3-PDO was favored when produced together with acetate, which was mostly the case in this study. Even if strong changes occurred in the microbial community structure over the pH range studied, high 1,3-PDO production yields were obtained and were comparable to the best yield obtained in similar conditions (i.e., mixed culture, pure glycerin, and no additive such as yeast extract) of 0.69 mol/mol [7].

## Additional files

10.1186/s13068-016-0447-8 COD mass balance of batch tests operated at variable initial pH. Detailed COD mass balances of each batch test are presented in this additional table. COD mass balances were calculated from the metabolites composition measured after 3 days of fermentation (triplicate experiments).
10.1186/s13068-016-0447-8 Metabolites distribution (expressed in COD equivalent) measured after total substrate depletion in pH-controlled reactors (four replicates). Detailed final COD distributions as assessed through metabolites production are presented in this Figure. Results are normalized by the initial COD contained in the medium.

## Abbreviations

1,3-PDO: 1,3-propanediol
ADP/ATP: adenosine di/triphosphate
HRT: hydraulic retention time
NADH_2_/NAD^+^: nicotinamide adenine dinucleotide reduced/oxidized
PCA: principal component analysis
PTT: polytrimethylene terephthalate
qPCR: quantitative real-time polymerase chain reaction

## References

[1] Marchetti JM, Miguel VU, Errazu AF (2007). Possible methods for biodiesel production. Renew Sustain Energy Rev..

[2] OECD-FAO: Biofuels. In: OECD-FAO Agricultural Outlook 2011–2020. OECD Publishing, 2011.p. 77–94. http://www.fnsea.fr/media/64283/outlookfaoocde.pdf. Accessed 01 Oct 2015.

[3] Zeng A-P, Sabra W (2011). Microbial production of diols as platform chemicals: recent progresses. Curr Opin Biotechnol.

[4] Wilkens E, Ringel AK, Hortig D, Willke T, Vorlop KD (2012). High-level production of 1,3-propanediol from crude glycerol by *Clostridium butyricum* AKR102a. Appl Microbiol Biotechnol.

[5] Clomburg J, Gonzalez R (2013). Anaerobic fermentation of glycerol: a platform for renewable fuels and chemicals. Trends Biotechnol.

[6] Dietz D, Zeng AP (2014). Efficient production of 1,3-propanediol from fermentation of crude glycerol with mixed cultures in a simple medium. Bioprocess Biosyst Eng.

[7] Selembo PA, Perez JM, Lloyd WA, Logan BE (2009). Enhanced hydrogen and 1,3-propanediol production from glycerol by fermentation using mixed cultures. Biotechnol Bioeng.

[8] Liu B, Christiansen K, Parnas R, Xu Z, Li B (2013). Optimizing the production of hydrogen and 1,3-propanediol in anaerobic fermentation of biodiesel glycerol. Int J Hydrogen Energ..

[9] Temudo MF, Poldermans R, Kleerebezem R, van Loosdrecht MCM (2008). Glycerol fermentation by (open) mixed cultures: a chemostat study. Biotechnol Bioeng.

[10] Kanjilal B, Noshadi I, Bautista EJ, Srivastava R, Parnas RS (2014). Batch, design optimization, and DNA sequencing study for continuous 1,3-propanediol production from waste glycerol by a soil-based inoculum. Appl Microbiol Biotechnol.

[11] Samul D, Leja K, Grajek W (2014). Impurities of crude glycerol and their effect on metabolite production. Ann Microbiol.

[12] Paillet F, Silva-illanes F, Marone A, Tapia-Venegas E, Cabrol L. Improvement of hydrogen production from glycerol in micro-oxidative environment. Presented at ICH2P-2015-6. International Conference on Hydrogen Production, Oshawa. Canada: University of Ontario Institute of Technology (UOIT). http://prodinra.inra.fr/record/305010. Accessed 01 Oct 2015.

[13] Herbert D, Phipps PJ, Strange RE, Norris JR, Ribbons DW (1971). Chapter III Chemical Analysis of Microbial Cells. Methods in Microbiology.

[14] Zeng A, Biebl H, Schlieker H, Deckwer W (1993). Pathway analysis of glycerol fermentation by *Klebsiella pneumoniae*: regulation of reducing equivalent balance and product formation. Enzym Microb Technol..

[15] Wang Y, Qian PY (2009). Conservative fragments in bacterial 16S rRNA genes and primer design for 16S ribosomal DNA amplicons in metagenomic studies. PLoS ONE.

[16] Wéry N, Bru-Adan V, Minervini C, Delgénes JP, Garrelly L, Godon JJ (2008). Dynamics of *Legionella* spp. and bacterial populations during the proliferation of *L. pneumophila* in a cooling tower facility. Appl Environ Microbiol.

[17] Michelland RJ, Dejean S, Combes S, Fortun-Lamothe L, Cauquil L, Note CP (2009). StatFingerprints: a friendly graphical interface program for processing and analysis of microbial fingerprint profiles. Mol Ecol Resour..

[18] Schloss PD, Westcott SL, Ryabin T, Hall JR, Hartmann M, Hollister EB, Lesniewski RA, Oakley BB, Parks DH, Robinson CJ, Sahl JW, Stres B, Thallinger GG, Van Horn DJ, Weber CF (2009). Introducing mothur: open-source, platform-independent, community-supported software for describing and comparing microbial communities. Appl Environ Microbiol.

[19] Ja Klappenbach, Saxman PR, Cole JR, Schmidt TM (2001). rrndb: the ribosomal RNA operon copy number database. Nucleic Acids Res..

[20] Bastidas-Oyanedel JR. Thermodynamic based modelling of biohydrogen production by anaerobic fermentation. PhD thesis, Université de Montpellier II, France. 2011.

[21] Gallardo R, Faria C, Rodrigues LR, Pereira MA, Alves MM (2014). Anaerobic granular sludge as a biocatalyst for 1,3-propanediol production from glycerol in continuous bioreactors. Bioresour Technol.

[22] Lee W-K, Fujisawa T, Kawamura S, Itoh K, Mitsuoka T (1989). *Clostridium intestinalis* sp. nov., an aerotolerant species isolated from the feces of cattle and pigs. Int J Syst Bacteriol.

[23] Gößner AS, Küsel K, Schulz D, Trenz S, Acker G, Lovell CR, Drake HL (2006). Trophic interaction of the aerotolerant anaerobe *Clostridium intestinale* and the acetogen *Sporomusa rhizae* sp. nov. isolated from roots of the black needlerush *Juncus roemerianus*. Microbiology.

[24] Lal S, Ramachandran U, Zhang X, Sparling R, Levin B (2013). Draft genome sequence of the hydrogen- and ethanol-producing bacterium Clostridium intestinale strain. URNW.

[25] Lee CS, Aroua MK, Daud WMAW, Cognet P, Pérès-Lucchese Y, Fabre PL, Reynes O, Latapie L (2015). A review: conversion of bioglycerol into 1,3-propanediol via biological and chemical method. Renew Sustain Energy Rev.

[26] Drożdżyńska A, Pawlicka J, Kubiak P, Kośmider A, Pranke D, Olejnik-Schmidt A, Czaczyk K (2014). Conversion of glycerol to 1,3-propanediol by *Citrobacter freundii* and *Hafnia alvei*—newly isolated strains from the *Enterobacteriaceae*. New Biotechnol.

[27] Rafrafi Y, Trably E, Hamelin J, Latrille E, Meynial-Salles I, Benomar S, Giudici-Orticoni MT, Steyer JP (2013). Sub-dominant bacteria as keystone species in microbial communities producing bio-hydrogen. Int J Hydrogen Energ..

[28] Temudo MF, Kleerebezem R, Van Loosdrecht M (2007). Influence of the pH on (open) mixed culture fermentation of glucose: a chemostat study. Biotechnol Bioeng.

[29] Rodríguez J, Kleerebezem R, Lema JM, Van Loosdrecht MCM (2006). Modeling product formation in anaerobic mixed culture fermentations. Biotechnol Bioeng.

[30] Suzuki T, Nishikawa C, Seta K, Shigeno T, Nakajima-Kambe T (2014). Ethanol production from glycerol-containing biodiesel waste by *Klebsiella variicola* shows maximum productivity under alkaline conditions. New Biotechnol.

[31] Millat T, Janssen H, Bahl H, Fischer R, Wolkenhauer O. The pH-induced metabolic shift from acidogenesis to solventogenesis in *Clostridium acetobutylicum—*From experiments to models. Beilstein-Institut 2011:33–54.

[32] Salakkam A, Webb C (2015). The inhibition effect of methanol, as a component of crude glycerol, on the growth rate of *Cupriavidus necator* and other micro-organisms. Biochem Eng J.

[33] Szymanowska-Powałowska D, Leja K (2014). An increasing of the efficiency of microbiological synthesis of 1,3-propanediol from crude glycerol by the concentration of biomass. Electron J Biotechnol.

[34] Szymanowska-Powałowska D (2015). The effect of high concentrations of glycerol on the growth, metabolism and adaptation capacity of *Clostridium butyricum* DSP1. Electron J Biotechnol.

[35] Xafenias N, Anunobi MO, Mapelli V (2015). Electrochemical startup increases 1,3-propanediol titers in mixed-culture glycerol fermentations. Process Biochem.

