# Supplementary material for: Consistent 1,3-propanediol production from glycerol in mixed culture fermentation over a wide range of pH
Source: Biotechnol Biofuels. 2016 Feb 6;9:32. doi: 10.1186/s13068-016-0447-8 (PMC4744455; doi:10.1186/s13068-016-0447-8)
Supplement: Supplementary file 1 — 10.1186/s13068-016-0447-8 COD mass balance of batch tests operated at variable initial pH. Detailed COD mass balances of each batch test are presented in this additional table. COD mass balances were calculated from the metabolites composition measured after 3 days of fermentation (triplicate experiments). [file 13068_2016_447_MOESM1_ESM.pdf]

Table S1 : COD mass balance of batch tests operated at variable initial pH. COD mass balances were calculated from the metabolites composition measured after 3 days of fermentation (triplicate experiments).

Initial pH	Measured end-products of glycerol fermentation (% initial COD)							Estimated biomass
	Glycerol	1,3-propanediol	Acetate	Lactate	Ethanol	Formate	H_2	
4	95.37±2.23	0	0	0	0	0	0	0
5	8.07±1.38	64.85 ±1.25	11.47 ±0.28	0	6.08 ±2.18	0	0.72 ±0.19	6.10 ±0.14
6	0	60.10 ±0.60	11.95 ±0.22	5.43 ±0.41	9.22 ±3.41	0	1.00 ±0.02	7.00 ±0.35
7	0	73.68 ±0.27	15.70 ±0.26	1.04 ±0.45	0	0	0.21 ±0.14	6.67 ±0.08
8	0	72.78 ±0.17	16.73 ±0.60	0	0	2.50 ±0.06		6.96 ±0.23
9	0	66.64 ±0.25	14.10 ±1.17	5.63 ±0.08	0	3.79 ±0.14		6.64 ±0.46
10	92.98 ±0.85	0	0	0	0	0	0	0
