# Supplementary material for: Consistent 1,3-propanediol production from glycerol in mixed culture fermentation over a wide range of pH
Source: Biotechnol Biofuels. 2016 Feb 6;9:32. doi: 10.1186/s13068-016-0447-8 (PMC4744455; doi:10.1186/s13068-016-0447-8)
Supplement: Supplementary file 2 — 10.1186/s13068-016-0447-8 Metabolites distribution (expressed in COD equivalent) measured after total substrate depletion in pH-controlled reactors (four replicates). Detailed final COD distributions as assessed through metabolites production are presented in this Figure. Results are normalized by the initial COD contained in the medium. [file 13068_2016_447_MOESM2_ESM.pdf]

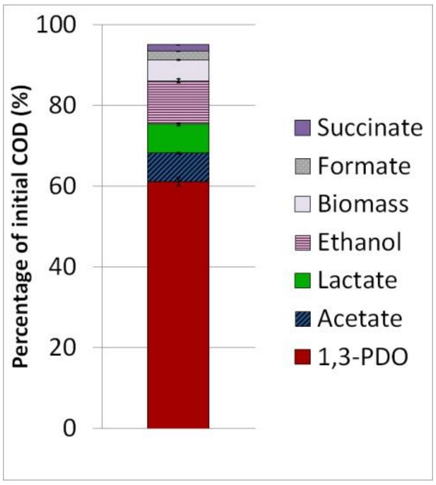

Figure S1 : Metabolites distribution (expressed in COD equivalent) measured after total substrate depletion in pH-controlled reactors (four replicated experiments). Results are normalized by the initial COD contained in the medium. The biomass was estimated from the theoretical ATP produced by the different metabolic pathways.
